# 6,6,9a-Trimethyl-5,5a,6,7,8,9,9a,9b-octa­hydro­naphtho[1,2-*c*]furan-1(3*H*)-one

**DOI:** 10.1107/S1600536808007460

**Published:** 2008-03-29

**Authors:** Iván Brito, Matías López-Rodríguez, Miguel Zárraga, Cristian Paz, Claudia Pérez

**Affiliations:** a Departamento de Química, Facultad de Ciencias Básicas, Universidad de Antofagasta, Casilla 170, Antofagasta, Chile; b Instituto de Bio-Orgánica ’Antonio González’, Universidad de La Laguna, Astrofísico Francisco Sánchez No. 2, La Laguna, Tenerife, Spain; c Departamento de Química Orgánica, Facultad de Ciencias Químicas, Universidad de Concepción, Casilla 160-C, Concepción, Chile; d Laboratorio de Fitoquímica, Facultad de Ciencias Biológicas, Universidad de Concepción y Centro de Investigación de Ecosistemas de la Patagonia (CIEP), Bilbao 449, Coyhaique, Chile

## Abstract

In the crystal structure of the title compound, C_15_H_22_O_2_, the cyclo­hexene and cyclo­hexane rings adopt half-boat and chair conformations, respectively, and the lactone ring is in an envelope conformation.

## Related literature

For related literature, see: Almeida *et al.* (2001 ▶); Appel *et al.* (1963 ▶); Cremer & Pople (1975 ▶); Cruz *et al.* (1973 ▶); Harinantenaina *et al.* (2007 ▶); Sierra *et al.* (1986 ▶).
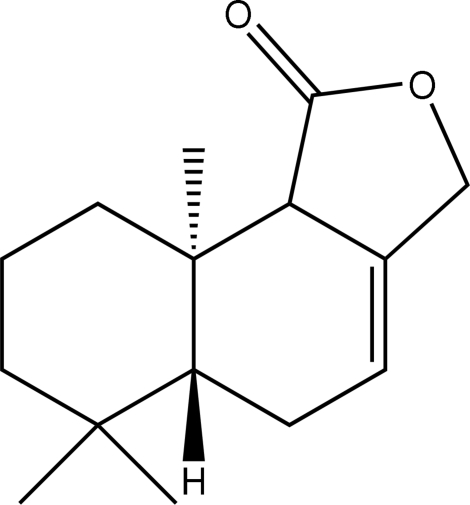


## Experimental

### 

#### Crystal data

C_15_H_22_O_2_
*M* *_r_* = 234.33
Orthorhombic, 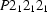

*a* = 7.4031 (2) Å
*b* = 7.9250 (2) Å
*c* = 22.9973 (8) Å
*V* = 1349.24 (7) Å^3^
*Z* = 4
Mo *K*α radiation
μ = 0.07 mm^−1^
*T* = 298 (2) K
0.14 × 0.12 × 0.08 mm

#### Data collection

Nonius KappaCCD area-detector diffractometer
Absorption correction: none
4453 measured reflections
1790 independent reflections
1645 reflections with *I* > 2σ(*I*)
*R*_int_ = 0.066

#### Refinement

*R*[*F* ^2^ > 2σ(*F* ^2^)] = 0.061
*wR*(*F* ^2^) = 0.167
*S* = 1.18
1790 reflections
163 parameters
H atoms treated by a mixture of independent and constrained refinement
Δρ_max_ = 0.23 e Å^−3^
Δρ_min_ = −0.19 e Å^−3^

### 

Data collection: *COLLECT* (Nonius, 1998 ▶); cell refinement: *DENZO–SMN* (Otwinowski & Minor, 1997 ▶); data reduction: *DENZO–SMN*; program(s) used to solve structure: *SIR97* (Altomare *et al.*, 1999 ▶); program(s) used to refine structure: *SHELXL97* (Sheldrick, 2008 ▶); molecular graphics: *ORTEP-3 for Windows* (Farrugia, 1997 ▶) and *PLATON* (Spek, 2003 ▶); software used to prepare material for publication: *WinGX* (Farrugia, 1999 ▶).

## Supplementary Material

Crystal structure: contains datablocks global, I. DOI: 10.1107/S1600536808007460/nc2094sup1.cif

Structure factors: contains datablocks I. DOI: 10.1107/S1600536808007460/nc2094Isup2.hkl

Additional supplementary materials:  crystallographic information; 3D view; checkCIF report

## Figures and Tables

**Fig. 1.**
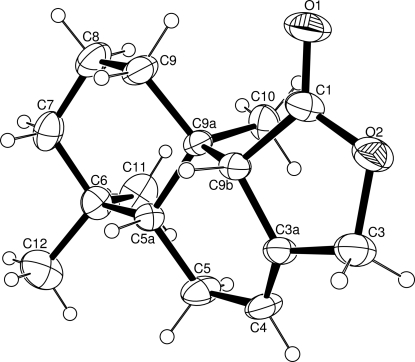
A view of the molecular structure of (I), showing the atom-numbering scheme. Displacement ellipsoids are drawn at the 30% probability level and H atoms are shown as small spheres of arbitrary radii.

## supplementary crystallographic information

### Comment

Drimys winteri J.*R*. Forst is a plant used in folk medicine of many Latinoamerican countries. In Chile, Drimys winteri (canelo) is used by the indigenous Mapuche in the treatment of several stomachal diseases, ulcers and hemorrhages (Almeida *et al.*,  2001). Chemical studies has shown the presence of a variety of sesquiterpenes with drimano skeleton (Appel *et al.*,  1963) and flavonoids. Some of these compounds have shown significant antibacterial, antifungi, antitumor and insecticide properties (Cruz *et al.*,  1973; Sierra *et al.*,  1986). The extract of Drimys winteri leaves afforded Cinnamolide and Drimenin two lactones with drimano skeleton. The title compound (I) is a positional isomer of Cinnamolide [IUPAC name: 6,6,9a-trimethyl-5,5a,6,7,8,9,9a,9 b-octahydronaphtho [1,2-*c*]furan-3(1*H*)-one] (CSD refcode NIDJUG; Harinantenaina *et al.*,  2007).In order to ascertain the structure and secure the assignment of the stereochemistry of (I) an X-ray analysis was performed but the absolute configuration was not determined by this analysis. The structure consists of a drimane skeleton and the methyl group at C9a is α -oriented. The cyclohexene ring (A) and cyclohexane ring (B) is in a half-boat and a chair conformation, respectively [Q_T_ = 0.526 (3) Å φ_2_ = 316.5 (4) °, q_2_ = 0.413 (3)Å for ring A; Q_T_ = 0.545 (3) Å, φ_2_ = 160 (4)°, q_2_ = 0.052 (4) Å for ring B], and the lactone ring is in an envelope conformation [q_2_=0.233 (3) Å, φ_2_ = 284.5 (7)°] (Cremer & Pople, 1975). The A and B rings are *trans*-fused.

### Experimental

*Drimys winteri *was collected from the Estuary of Reloncaví, *X*^th°^ Región, Chile in November 2005. Two kilograms of bark was extracted in dichloromethane and concentrated by rotavapor to yield 180 g. 30 grams of crude extract was subjected to flash chromatography on Silicagel G, 70–200 mesh with hexane–ethyl-acetate mixtures of increasing polarity as elution solvents. Pure components were obtained by further chromatography on silicagel of the fraction 10% hexane–ethyl-acetate (11 g). Recrystallization from methanol,at room temperature afforded colourless crystals of drimenin (0.02 g) suitable for X-ray difracction analysis. NMR spectra (^1^H-RMN, ^13^C-RMN, DEPT and ^1^H-^1^H COSY) were obtained on a Bruker AC 250P multinuclear spectrometer, in DCCl_3_ with TMS as internal standard. Drimenin(C_15_H_22_O_2_); Colorless crystals, mp 95 - 97°C. ^1^H-RMN (250 MHz) δ(p.p.m.); 0.88(3*H*, s), 0.90 (3*H*, s), 0.92 (3*H*, s), 1.15–1.30 (2*H*, m), 1.35 (1*H*, dd, J=3.4, 5.0, 13 Hz), 2.77 (1*H*, br s), 4.65 (2*H*, m), 5.73 (1*H*, br s). ^13^C-RMN δ(p.p.m.) 175.3 (*s*); 121.1 (*d*); 129.8 (*s*); 69.8 (*t*); 53.6 (*d*); 49.6 (*d*); 42.3 (*t*); 38.4 (*t*); 34.3 (*s*); 33.0 (*q*); 31.1 (*s*); 23.3 (*t*); 21.4 (*q*); 18.3 (*t*); 13.9 (*q*).

### Refinement

The H atom bonded to C4 was found in difference maps and was freely refined. All other H atoms were positioned with idealized geometry (C—H = 0.96–0.98 Å) and were refined using a riding model, with *U*_iso_(H) = 1.2*U*_eq_(C) (1.5*U*_eq_ for methyl H atoms) of the carrier atom. In the absence of any significant anomalous scattering, Friedel equivalents were merged prior to the final refinements, and the absolute structure was not determined.

### Crystal data

**Table:**

C_15_H_22_O_2_	*F*_000_ = 512
*M**_r_* = 234.33	*D*_x_ = 1.154 Mg m^−^^3^
Orthorhombic, *P*2_1_2_1_2_1_	Mo *K*α radiation λ = 0.71073 Å
Hall symbol: P 2ac 2ab	Cell parameters from 4453 reflections
*a* = 7.4031 (2) Å	θ = 2.7–27.5º
*b* = 7.9250 (2) Å	µ = 0.07 mm^−^^1^
*c* = 22.9973 (8) Å	*T* = 298 (2) K
*V* = 1349.24 (7) Å^3^	Prism, colourless
*Z* = 4	0.14 × 0.12 × 0.08 mm

### Data collection

**Table:**

Nonius KappaCCD area-detector diffractometer	1645 reflections with *I* > 2σ(*I*)
Radiation source: fine-focus sealed tube	*R*_int_ = 0.066
Monochromator: graphite	θ_max_ = 27.5º
φ scans, and ω scans with κ offsets	θ_min_ = 2.7º
Absorption correction: none	*h* = −9→9
4453 measured reflections	*k* = −10→10
1790 independent reflections	*l* = −29→29

### Refinement

**Table:**

Refinement on *F*^2^	H atoms treated by a mixture of independent and constrained refinement
Least-squares matrix: full	*w* = 1/[σ^2^(*F*_o_^2^) + (0.0906*P*)^2^ + 0.1776*P*] where *P* = (*F*_o_^2^ + 2*F*_c_^2^)/3
*R*[*F*^2^ > 2σ(*F*^2^)] = 0.061	(Δ/σ)_max_ = 0.001
*wR*(*F*^2^) = 0.167	Δρ_max_ = 0.23 e Å^−^^3^
*S* = 1.18	Δρ_min_ = −0.19 e Å^−^^3^
1790 reflections	Extinction correction: none
163 parameters	

### Special details

**Table:**

Geometry. All e.s.d.'s (except the e.s.d. in the dihedral angle between two l.s. planes) are estimated using the full covariance matrix. The cell e.s.d.'s are taken into account individually in the estimation of e.s.d.'s in distances, angles and torsion angles; correlations between e.s.d.'s in cell parameters are only used when they are defined by crystal symmetry. An approximate (isotropic) treatment of cell e.s.d.'s is used for estimating e.s.d.'s involving l.s. planes.
Refinement. Refinement of *F*^2^ against ALL reflections. The weighted *R*-factor *wR* and goodness of fit *S* are based on *F*^2^, conventional *R*-factors *R* are based on *F*, with *F* set to zero for negative *F*^2^. The threshold expression of *F*^2^ > σ(*F*^2^) is used only for calculating *R*-factors(gt) *etc*. and is not relevant to the choice of reflections for refinement. *R*-factors based on *F*^2^ are statistically about twice as large as those based on *F*, and *R*- factors based on ALL data will be even larger.

### Fractional atomic coordinates and isotropic or equivalent isotropic displacement parameters (Å^2^)

**Table:**

	*x*	*y*	*z*	*U*_iso_*/*U*_eq_	
O1	0.8222 (4)	0.4735 (2)	0.79867 (11)	0.0726 (8)	
O2	0.6477 (3)	0.2766 (3)	0.76017 (10)	0.0656 (7)	
C1	0.7967 (4)	0.3267 (3)	0.78912 (12)	0.0513 (7)	
C3	0.6436 (5)	0.0949 (4)	0.75322 (14)	0.0609 (8)	
H3A	0.6714	0.0632	0.7135	0.077 (3)*	
H3B	0.5261	0.0499	0.7635	0.077 (3)*	
C3A	0.7862 (4)	0.0326 (3)	0.79414 (10)	0.0427 (6)	
C4	0.7941 (4)	−0.1115 (3)	0.82241 (13)	0.0537 (7)	
H4	0.712 (4)	−0.196 (4)	0.8180 (12)	0.052 (8)*	
C5	0.9382 (5)	−0.1455 (4)	0.86662 (14)	0.0607 (8)	
H5A	0.9938	−0.2535	0.858	0.077 (3)*	
H5B	0.8825	−0.1539	0.9047	0.077 (3)*	
C5A	1.0857 (4)	−0.0101 (3)	0.86874 (10)	0.0400 (5)	
H5A1	1.1546	−0.0261	0.8328	0.077 (3)*	
C6	1.2257 (4)	−0.0397 (4)	0.91848 (12)	0.0533 (7)	
C7	1.3670 (4)	0.1013 (5)	0.91648 (15)	0.0648 (8)	
H7A	1.4451	0.0906	0.9501	0.077 (3)*	
H7B	1.4411	0.086	0.8821	0.077 (3)*	
C8	1.2894 (5)	0.2776 (5)	0.91553 (17)	0.0701 (9)	
H8A	1.3868	0.3592	0.9131	0.077 (3)*	
H8B	1.2234	0.2982	0.9513	0.077 (3)*	
C9	1.1637 (4)	0.2991 (4)	0.86386 (14)	0.0573 (7)	
H9A	1.2326	0.2857	0.8283	0.077 (3)*	
H9B	1.1148	0.4126	0.8642	0.077 (3)*	
C9A	1.0067 (3)	0.1719 (3)	0.86379 (10)	0.0383 (5)	
C9B	0.9142 (3)	0.1759 (3)	0.80383 (10)	0.0391 (5)	
H9B1	1.009	0.1684	0.7742	0.077 (3)*	
C10	0.8694 (4)	0.2156 (4)	0.91074 (12)	0.0587 (8)	
H10A	0.9297	0.2262	0.9475	0.100 (5)*	
H10B	0.7804	0.1278	0.9131	0.100 (5)*	
H10C	0.8114	0.3204	0.9012	0.100 (5)*	
C11	1.1446 (6)	−0.0480 (6)	0.97981 (13)	0.0809 (11)	
H11A	1.0476	−0.1284	0.9804	0.100 (5)*	
H11B	1.0992	0.0612	0.9905	0.100 (5)*	
H11C	1.2361	−0.0819	1.007	0.100 (5)*	
C12	1.3245 (6)	−0.2074 (5)	0.90694 (18)	0.0871 (12)	
H12A	1.4199	−0.2213	0.9348	0.100 (5)*	
H12B	1.3744	−0.2062	0.8684	0.100 (5)*	
H12C	1.2406	−0.2993	0.9104	0.100 (5)*	

### Atomic displacement parameters (Å^2^)

**Table:**

	*U*^11^	*U*^22^	*U*^33^	*U*^12^	*U*^13^	*U*^23^
O1	0.0919 (18)	0.0321 (10)	0.0938 (17)	0.0012 (11)	−0.0179 (15)	−0.0003 (9)
O2	0.0735 (13)	0.0419 (11)	0.0814 (14)	0.0128 (10)	−0.0286 (13)	−0.0048 (9)
C1	0.0645 (17)	0.0336 (13)	0.0558 (15)	0.0033 (13)	−0.0039 (14)	−0.0002 (10)
C3	0.0734 (19)	0.0432 (15)	0.0660 (18)	0.0044 (14)	−0.0219 (17)	−0.0077 (12)
C3A	0.0499 (14)	0.0330 (12)	0.0450 (13)	0.0001 (11)	−0.0071 (12)	−0.0062 (9)
C4	0.0597 (17)	0.0348 (12)	0.0667 (17)	−0.0153 (12)	−0.0151 (15)	−0.0006 (12)
C5	0.0749 (19)	0.0385 (14)	0.0686 (18)	−0.0126 (14)	−0.0193 (17)	0.0144 (12)
C5A	0.0446 (12)	0.0372 (12)	0.0384 (11)	−0.0012 (11)	−0.0037 (10)	0.0012 (9)
C6	0.0537 (15)	0.0590 (16)	0.0473 (14)	0.0056 (14)	−0.0096 (13)	0.0042 (12)
C7	0.0441 (15)	0.091 (2)	0.0595 (17)	−0.0051 (16)	−0.0102 (15)	−0.0033 (16)
C8	0.0543 (16)	0.072 (2)	0.084 (2)	−0.0206 (16)	−0.0167 (17)	−0.0073 (17)
C9	0.0545 (16)	0.0464 (15)	0.0709 (17)	−0.0171 (14)	−0.0049 (15)	0.0000 (13)
C9A	0.0395 (11)	0.0348 (11)	0.0405 (11)	−0.0083 (10)	0.0023 (10)	−0.0025 (9)
C9B	0.0453 (12)	0.0311 (11)	0.0410 (11)	−0.0017 (11)	0.0025 (10)	−0.0006 (9)
C10	0.0536 (15)	0.075 (2)	0.0476 (15)	0.0070 (15)	0.0044 (13)	−0.0152 (14)
C11	0.084 (2)	0.112 (3)	0.0464 (16)	−0.013 (3)	−0.0105 (17)	0.0190 (17)
C12	0.094 (3)	0.079 (2)	0.088 (3)	0.029 (2)	−0.040 (2)	−0.0022 (19)

### Geometric parameters (Å, °)

**Table:**

O1—C1	1.199 (3)	C7—H7A	0.97
O2—C1	1.348 (4)	C7—H7B	0.97
O2—C3	1.449 (4)	C8—C9	1.519 (5)
C1—C9B	1.516 (3)	C8—H8A	0.97
C3—C3A	1.498 (4)	C8—H8B	0.97
C3—H3A	0.97	C9—C9A	1.538 (3)
C3—H3B	0.97	C9—H9A	0.97
C3A—C4	1.315 (4)	C9—H9B	0.97
C3A—C9B	1.496 (3)	C9A—C10	1.523 (4)
C4—C5	1.498 (4)	C9A—C9B	1.540 (3)
C4—H4	0.91 (3)	C9B—H9B1	0.98
C5—C5A	1.532 (4)	C10—H10A	0.96
C5—H5A	0.97	C10—H10B	0.96
C5—H5B	0.97	C10—H10C	0.96
C5A—C9A	1.560 (3)	C11—H11A	0.96
C5A—C6	1.561 (3)	C11—H11B	0.96
C5A—H5A1	0.98	C11—H11C	0.96
C6—C7	1.531 (4)	C12—H12A	0.96
C6—C11	1.534 (4)	C12—H12B	0.96
C6—C12	1.540 (5)	C12—H12C	0.96
C7—C8	1.511 (5)		
			
C1—O2—C3	111.4 (2)	C7—C8—H8A	109.6
O1—C1—O2	120.4 (3)	C9—C8—H8A	109.6
O1—C1—C9B	129.3 (3)	C7—C8—H8B	109.6
O2—C1—C9B	110.3 (2)	C9—C8—H8B	109.6
O2—C3—C3A	104.1 (2)	H8A—C8—H8B	108.1
O2—C3—H3A	110.9	C8—C9—C9A	113.0 (2)
C3A—C3—H3A	110.9	C8—C9—H9A	109
O2—C3—H3B	110.9	C9A—C9—H9A	109
C3A—C3—H3B	110.9	C8—C9—H9B	109
H3A—C3—H3B	108.9	C9A—C9—H9B	109
C4—C3A—C9B	123.9 (2)	H9A—C9—H9B	107.8
C4—C3A—C3	128.9 (3)	C10—C9A—C9	110.8 (2)
C9B—C3A—C3	106.8 (2)	C10—C9A—C9B	109.5 (2)
C3A—C4—C5	121.6 (2)	C9—C9A—C9B	108.9 (2)
C3A—C4—H4	123.5 (19)	C10—C9A—C5A	114.1 (2)
C5—C4—H4	114.9 (19)	C9—C9A—C5A	108.8 (2)
C4—C5—C5A	113.8 (2)	C9B—C9A—C5A	104.53 (18)
C4—C5—H5A	108.8	C3A—C9B—C1	101.6 (2)
C5A—C5—H5A	108.8	C3A—C9B—C9A	113.53 (19)
C4—C5—H5B	108.8	C1—C9B—C9A	118.1 (2)
C5A—C5—H5B	108.8	C3A—C9B—H9B1	107.7
H5A—C5—H5B	107.7	C1—C9B—H9B1	107.7
C5—C5A—C9A	112.2 (2)	C9A—C9B—H9B1	107.7
C5—C5A—C6	113.0 (2)	C9A—C10—H10A	109.5
C9A—C5A—C6	116.2 (2)	C9A—C10—H10B	109.5
C5—C5A—H5A1	104.7	H10A—C10—H10B	109.5
C9A—C5A—H5A1	104.7	C9A—C10—H10C	109.5
C6—C5A—H5A1	104.7	H10A—C10—H10C	109.5
C7—C6—C11	109.1 (3)	H10B—C10—H10C	109.5
C7—C6—C12	107.5 (3)	C6—C11—H11A	109.5
C11—C6—C12	107.9 (3)	C6—C11—H11B	109.5
C7—C6—C5A	108.8 (2)	H11A—C11—H11B	109.5
C11—C6—C5A	114.8 (3)	C6—C11—H11C	109.5
C12—C6—C5A	108.6 (2)	H11A—C11—H11C	109.5
C8—C7—C6	114.5 (2)	H11B—C11—H11C	109.5
C8—C7—H7A	108.6	C6—C12—H12A	109.5
C6—C7—H7A	108.6	C6—C12—H12B	109.5
C8—C7—H7B	108.6	H12A—C12—H12B	109.5
C6—C7—H7B	108.6	C6—C12—H12C	109.5
H7A—C7—H7B	107.6	H12A—C12—H12C	109.5
C7—C8—C9	110.4 (3)	H12B—C12—H12C	109.5
			
C3—O2—C1—O1	179.8 (3)	C8—C9—C9A—C9B	−167.1 (3)
C3—O2—C1—C9B	1.3 (3)	C8—C9—C9A—C5A	−53.7 (3)
C1—O2—C3—C3A	13.5 (4)	C5—C5A—C9A—C10	57.9 (3)
O2—C3—C3A—C4	150.1 (3)	C6—C5A—C9A—C10	−74.3 (3)
O2—C3—C3A—C9B	−23.0 (3)	C5—C5A—C9A—C9	−177.8 (2)
C9B—C3A—C4—C5	−1.5 (5)	C6—C5A—C9A—C9	50.0 (3)
C3—C3A—C4—C5	−173.5 (3)	C5—C5A—C9A—C9B	−61.6 (3)
C3A—C4—C5—C5A	−8.2 (4)	C6—C5A—C9A—C9B	166.2 (2)
C4—C5—C5A—C9A	41.4 (3)	C4—C3A—C9B—C1	−150.4 (3)
C4—C5—C5A—C6	175.2 (3)	C3—C3A—C9B—C1	23.0 (3)
C5—C5A—C6—C7	179.6 (3)	C4—C3A—C9B—C9A	−22.6 (4)
C9A—C5A—C6—C7	−48.5 (3)	C3—C3A—C9B—C9A	150.9 (2)
C5—C5A—C6—C11	−57.9 (4)	O1—C1—C9B—C3A	166.3 (3)
C9A—C5A—C6—C11	74.0 (3)	O2—C1—C9B—C3A	−15.4 (3)
C5—C5A—C6—C12	63.0 (3)	O1—C1—C9B—C9A	41.4 (4)
C9A—C5A—C6—C12	−165.2 (3)	O2—C1—C9B—C9A	−140.3 (2)
C11—C6—C7—C8	−74.4 (3)	C10—C9A—C9B—C3A	−71.3 (3)
C12—C6—C7—C8	168.9 (3)	C9—C9A—C9B—C3A	167.5 (2)
C5A—C6—C7—C8	51.5 (3)	C5A—C9A—C9B—C3A	51.4 (3)
C6—C7—C8—C9	−57.5 (4)	C10—C9A—C9B—C1	47.6 (3)
C7—C8—C9—C9A	58.3 (4)	C9—C9A—C9B—C1	−73.7 (3)
C8—C9—C9A—C10	72.5 (3)	C5A—C9A—C9B—C1	170.2 (2)
